# Does receiving red yeast rice prescriptions (LipoCol Forte^®^) increase the risk of end-stage renal disease or not? A retrospective cohort study

**DOI:** 10.1371/journal.pone.0345243

**Published:** 2026-10-27

**Authors:** Chuen-Chau Chang, Fai Lam, Chun-Chieh Yeh, Jaung-Geng Lin, Ta-Liang Chen, Chien-Chang Liao

**Affiliations:** 1 Department of Anesthesiology, Taipei Medical University Hospital, Taiwan; 2 Anesthesiology and Health Policy Research Center, Taipei Medical University Hospital, Taiwan; 3 Department of Anesthesiology, School of Medicine, College of Medicine, Taipei Medical University, Taiwan; 4 Department of Surgery, China Medical University Hospital, Taichung, Taiwan; 5 Department of Surgery, University of Illinois, Chicago, Illinois, United States of America; 6 School of Chinese Medicine, College of Chinese Medicine, China Medical University, Taiwan; 7 Department of Anesthesiology, Wan Fang Hospital, Taipei Medical University, Taiwan; 8 Research Center of Big Data and Meta-Analysis, Wan Fang Hospital, Taipei Medical University, Taipei, Taiwan; Universiti Tunku Abdul Rahman Fakulti Perubatan dan Sains Kesihatan M Kandiah, MALAYSIA

## Abstract

**Background:**

The potential relationship between the consumption of red yeast rice (RYR) and the development of end-stage renal disease (ESRD) remains poorly understood. We aimed to evaluate the risk of ESRD in patients who used RYR.

**Methods:**

Utilizing insurance claim records from 2010 to 2014, we performed a retrospective cohort analysis comparing 67,876 individuals using RYR against a control group of 90,836 lovastatin users (non-RYR group), with follow-up extending through 2019. The incidence of ESRD was analyzed using adjusted hazard ratios (HRs) and 95% confidence intervals (CIs) within Cox proportional hazard models.

**Results:**

During follow-up, the ESRD incidence was 0.9 per 1,000 person-years in RYR users, compared to 3.7 per 1,000 person-years in lovastatin users (HR 0.36, 95% CI 0.32–0.40). A dose-dependent reduction in ESRD risk was observed with increasing RYR prescriptions. Sensitivity analysis, excluding ESRD cases within the initial six months, confirmed a persistent association between RYR use and reduced ESRD risk (adjusted HR 0.72, 95% CI 0.64–0.81).

**Conclusion:**

RYR intake may be linked to a decreased likelihood of progressing to ESRD. Despite these promising results, factors such as medication adherence necessitate cautious interpretation. Further randomized controlled trials are required to validate these findings and clarify RYR’s role in slowing kidney disease progression.

## Introduction

Clinical observations emerging from Japan in early 2024 pointed to various health issues among consumers of Benikoji CholesteHelp^®^, a dietary supplement containing red yeast rice [1–5]. Statistical data updated by April 17, 2024, indicated that 236 patients required inpatient care for severe renal impairment potentially associated with this product. Evidence from clinical case studies showed that patients suffering from acute tubulointerstitial nephritis experienced a swift recovery upon the cessation of the supplement and the administration of prednisolone therapy [4]. Furthermore, multiple reports documented the development of Fanconi syndrome in individuals after they ingested Benikoji CholesteHelp^®^ [2,3]. Official investigations by the Japanese authorities eventually connected 76 deaths to this health crisis. Scientific analysis suggests that puberulic acid identified within the contaminated supplement batches may act as the primary trigger for these diverse renal pathologies including kidney failure and Fanconi syndrome.

Red yeast rice (RYR), produced through the fermentation of Monascus purpureus Went rice, contains the active compound monacolin K and is utilized extensively throughout Asia as a dietary supplement, a coloring agent, and a component of traditional Chinese medicine (TCM) [6–9]. Within clinical practice, physicians often prescribe standardized RYR preparations like LipoCol Forte^®^, HypoCol, and Xuezhikang to address hyperlipidemia [10–15]. Results from various clinical trials have verified that RYR effectively enhances lipid profiles by reducing total cholesterol along with triglycerides and low density lipoprotein cholesterol [7,13,14,16–18]. Furthermore, some research has focused on its anti inflammatory properties and the potential for RYR to decrease mortality and complication risks in treated populations [19,20]. While the use of RYR is generally viewed as safe, it remains crucial to maintain persistent monitoring for any adverse reactions.

Although previous investigations have identified various adverse reactions related to RYR, clinical data regarding its potential to cause kidney damage or renal dysfunction remain limited. This research employs a broad population based database to analyze the occurrence and medical consequences of end stage renal disease (ESRD) in patients who received prescriptions for medical grade RYR, with a specific focus on the formulation known as LipoCol Forte^®^.

## Methods

### Source of data

Information for this research was obtained from a government administered academic database in Taiwan which originates from the national health insurance framework. We accessed the data for research purposes on 01/01/2025. Previous literature has thoroughly detailed the organization and reliability of this data source [10–12,15]. Following the protocols established by the Ministry of Health and Welfare in Taiwan, the requirement for informed consent was waived because all individual identities were deidentified and processed through secure encryption to ensure privacy. The protocol for this investigation was granted ethical clearance by the Institutional Review Board of Taipei Medical University under the reference numbers (TMU-JIRB-202303013; TMU-JIRB-202003097; TMU-JIRB-201905042; TMU-JIRB-201902053).

### Study design

Using a total population of 23 million insured residents, we executed a retrospective cohort analysis as illustrated in Fig 1. Our study population comprised 67,876 individuals who were prescribed red yeast rice, specifically LipoCol Forte^®^, alongside a comparison group of 90,836 patients who received lovastatin but had no history of RYR intake between January 1, 2010, and December 31, 2014. Both study groups included participants aged 20 or older with a diagnosis of hyperlipidemia, with follow-up observations continuing until December 31, 2019. Throughout this timeframe, person years were calculated for all subjects until the occurrence of an ESRD diagnosis necessitating dialysis or the point of censoring, which included death, exit from the insurance scheme, or loss to follow-up. The control cohort was restricted to individuals who never utilized RYR at any point during the observation window. There was no immortal time bias in this study because the follow-up started from the time of the use of medication (lovastatin or RYR) or the index date and lasted until the occurrence of ESRD, censoring due to death, migration, or loss of follow-up by the end of 2019. We evaluated the risk of incident ESRD between the RYR cohort and the non-RYR cohort (use of lovastatin) during the follow-up period.

**Fig 1:**
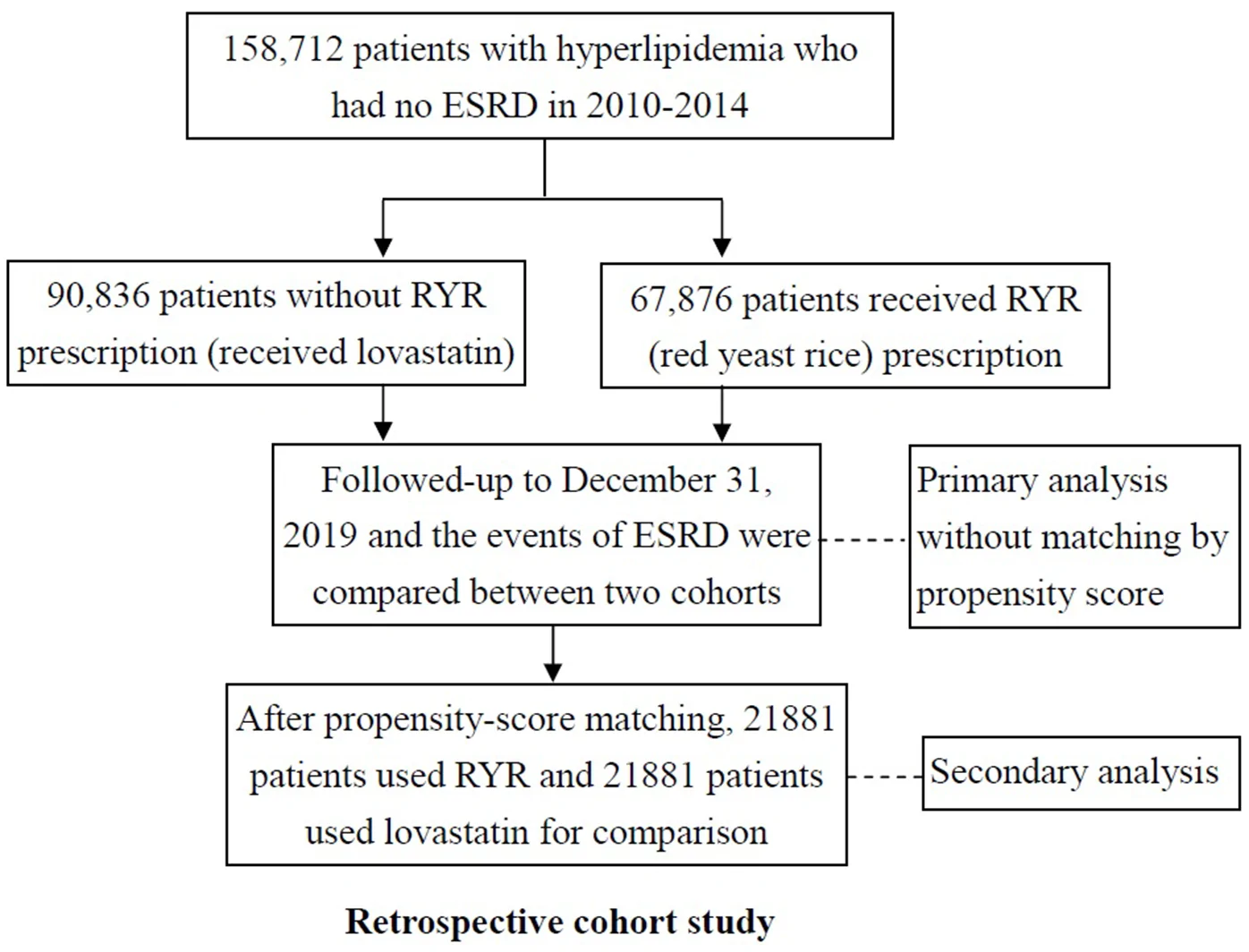
The selection process of adequate study subjects for the retrospective cohort study.

Regarding the concern on immortal time bias, it is important to clarify that  the index date was defined as the date of the first prescription of either RYR or lovastatin. By treating the treatment exposure as a time-varying covariate and initiating follow-up for both cohorts exactly at the onset of treatment (time-zero), we ensured that the period of observation and the period of exposure began simultaneously. This approach effectively eliminates immortal time bias by ensuring that no survival time was ‘guaranteed’ prior to the classification of exposure status. We evaluated the risk of incident ESRD between the RYR cohort and the non-RYR cohort starting from the index date, which was defined as the date of the first prescription for either medication. Because all patients were followed from their first treatment initiation, and the exposure status was determined based on the initial treatment decision, the impact of immortal time bias was minimized. This methodological approach is supported by statistical literature [21], which emphasizes that follow-up should begin at the same time as the treatment groups are defined to avoid bias.

### Measures and Criteria

Based on our earlier research [10–12,15], we examined the correlation between RYR consumption and ESRD risk while adjusting for various sociodemographic variables including age, gender, and low-income status. We also accounted for primary health conditions such as hyperlipidemia and other comorbidities including hypertension, diabetes, mental disorders, chronic obstructive pulmonary disease, ischemic heart disease, heart failure, and liver cirrhosis (S1 Table). The age of each subject was recorded at the initial date they received their first prescription for either RYR or lovastatin. In alignment with established methodologies for insurance data analysis, ESRD was defined as a primary clinical diagnosis (ICD-9-CM 585.6 and ICD-10-CM N18.6) coupled with the requirement for chronic renal replacement therapy such as peritoneal dialysis or hemodialysis (S1 Table). Hyperlipidemia was identified through at least one documented outpatient physician visit for the condition. Comprehensive information regarding the specific diagnostic codes used in this study is available in S1 Table.

To accurately identify RYR users in this study, we included individuals who visited TCM clinics and received physician-prescribed RYR under the Taiwan National Health Insurance program. The RYR prescribed in this cohort was LipoCol Forte^®^, a concentrated Chinese herbal medicine. Unlike conventional biochemical medications, LipoCol Forte^®^ is a natural herbal extract. For the purposes of this investigation, RYR was defined as the medical grade product LipoCol Forte^®^, which contains 600 mg of Monascus purpureus Went rice and 5.7 mg of lovastatin per capsule. Following the reimbursement criteria established by the National Health Insurance system, RYR was authorized for patients presenting with total cholesterol of 200 mg/dL or higher, low-density lipoprotein cholesterol of 130 mg/dL or higher, or triglyceride levels reaching at least 200 mg/dL. The standard therapeutic regimen involved the consumption of two capsules daily for a period of no less than one month. Adhering to validated research protocols, we categorized the RYR cohort as individuals who attended TCM clinics and obtained one or more prescriptions issued by a physician [10–12,15]. In the context of TCM clinical practice, each prescription for RYR generally provides a 30-day supply of the medication. Physicians prescribed RYR exclusively for dyslipidemia, the primary clinical indication for its use. Under Taiwan’s National Health Insurance regulations, the prescription of RYR is strictly restricted to this condition and is not indicated for other diseases.

Medications including angiotensin-converting enzyme inhibitors, angiotensin II receptor blockers, diuretics, and nephrotoxic agents (such as Gentamicin, Tobramycin, Vancomycin, Amikacin, and Cisplatin) were identified as factors associated with ESRD and were included as baseline characteristics to compare lovastatin and RYR users.

### Statistical analysis

The National Health Insurance’s evolution over the past three decades demonstrates the importance of continuous investment in data quality, privacy protection, and interoperability. The validity and reliability of disease diagnoses and medications have been evaluated with high quality in previous studies [22,23]. Due to the high quality of the data source and our specific inclusion criteria, no missing data were observed for demographic variables, comorbidities, or medication exposure. The National Health Insurance system covers over 99% of Taiwan’s population. In this study, the dataset was linked to the National Death Registry to ascertain mortality; thus, the likelihood of loss to follow-up due to death is virtually zero. Furthermore, any potential attrition due to emigration is expected to be balanced and comparable between the lovastatin and RYR groups.

Distributional differences in baseline demographics between the cohorts with and without RYR intake were evaluated using chi square tests. To determine the link between RYR prescriptions and ESRD risk, we applied multivariate Cox proportional hazards regression models, controlling for variables such as sociodemographic factors, pre existing medical conditions, and medications taken concurrently as shown in Table 1. During the observation window, adjusted hazard ratios (HRs) and 95% confidence intervals (CIs) were determined for ESRD incidence. We also performed stratified analyses to explore the correlation between RYR usage and ESRD likelihood across different subgroups. To address the concern about multiple testing, we have adjusted the significance levels using the Bonferroni correction. To ensure the reliability of our findings, a sensitivity analysis was executed by omitting ESRD events that occurred during the first 1–6 months of follow up.

**Table 1:** Baseline characteristics between cohorts with and without use of red yeast rice prescription.

	No RYR N = 90836		RYR prescription N = 67876		p-value
Sex	n	(%)	n	(%)	0.5681
Female	51445	(56.6)	38539	(56.8)	
Male	39391	(43.4)	29337	(43.2)	
Age, years					<0.0001
20-29	651	(0.7)	3016	(4.4)	
30-39	2935	(3.2)	9406	(13.9)	
40-49	10535	(11.6)	16576	(24.4)	
50-59	25407	(28.0)	22047	(32.5)	
60-69	25022	(27.6)	12223	(18.0)	
70-79	19245	(21.2)	3882	(5.7)	
≥80	7041	(7.8)	726	(1.1)	
Low income					<0.0001
No	86964	(95.7)	65609	(96.7)	
Yes	3872	(4.3)	2267	(3.3)	
Number of hospitalizations					<0.0001
0	27347	(30.1)	37295	(55.0)	
1	18038	(19.9)	14179	(20.9)	
2	12187	(13.4)	6686	(9.9)	
≥3	33264	(36.6)	9716	(14.3)	
Number of emergency visits					<0.0001
0	18684	(20.6)	24095	(35.5)	
1	16380	(18.0)	15409	(22.7)	
2	12732	(14.0)	9419	(13.9)	
≥3	43040	(47.4)	18953	(27.9)	
Medical conditions					
Hypertension	56453	(62.2)	26691	(39.3)	<0.0001
Diabetes	47873	(52.7)	16147	(23.8)	<0.0001
Mental disorders	41838	(46.1)	27243	(40.1)	<0.0001
COPD	13936	(15.3)	4432	(6.5)	<0.0001
Ischemic heart disease	25574	(28.2)	11500	(16.9)	<0.0001
Heart failure	7174	(7.9)	1603	(2.4)	<0.0001
Liver cirrhosis	6955	(7.7)	4572	(6.7)	<0.0001
Chronic kidney disease	31469	(34.6)	9766	(14.4)	<0.0001
CCI, score					<0.0001
0	8258	(9.1)	17747	(26.2)	
1	15119	(16.6)	13004	(19.2)	
2	13469	(14.8)	11980	(17.7)	
≥3	53990	(59.4)	25145	(37.1)	
Anticoagulant drug use	4763	(5.2)	1072	(1.6)	<0.0001
Anti-hypertension drug use	47916	(52.7)	20059	(29.5)	<0.0001
ACE inhibitors drug use	30839	(33.9)	7525	(11.1)	<0.0001
ARBs drug use	59422	(65.4)	25663	(37.8)	<0.0001
Nephrotoxic drugs use	23794	(26.2)	8950	(13.2)	<0.0001
Diuretics drugs use	44990	(49.5)	13923	(20.5)	<0.0001

CCI, Charlson comorbidity index; RYR, red yeast rice.

To account for the competing risk of all-cause mortality, the cumulative incidence of ESRD was estimated, and the associations were evaluated using the Fine-Gray subdistribution hazard model, which excludes all-cause death cases from the outcome events but retains them in the risk set during the follow-up period. To evaluation the dynamic impact of RYR on ESRD, we conducted a cumulative cohort analysis across different time period of foolow-up (Years 0–10). Incidence rates were expressed as events per 1,000 person-years. HRs and their corresponding 95% confidence intervals (CIs) were estimated for each annual cut-off point, utilizing the non-RYR cohort as the reference group. All computational processes were performed with SAS for Windows version 9.1 (SAS Institute Inc), where a two sided P value below 0.05 was defined as statistically significant.

## Results

Overall, a total of 90,836 patients with lovastatin prescriptions (non-RYR group) and 67,876 with RYR prescriptions were included in the analysis (Fig 1). As shown in Table 1, participants in the RYR cohort had lower proportions of older individuals (p < 0.0001), low-income status (p < 0.0001), hospital admissions (p < 0.0001), emergency department visits (p < 0.0001), and underlying medical conditions (p < 0.0001) compared to the non-RYR cohort. Additionally, individuals in the RYR group exhibited lower proportions of Charlson Comorbidity Index scores of ≥3, anticoagulant use (p < 0.0001), and antihypertensive medication use (p < 0.0001) than those in the non-RYR group.

During the follow-up period (Table 2), the incidence of end-stage renal disease (ESRD) was 0.9 per 1,000 person-years in patients prescribed RYR, compared to 3.7 per 1,000 person-years in those who did not receive RYR. After adjusting for the baseline characteristics listed in Table 1, the use of RYR was significantly associated with a reduced risk of ESRD, with an adjusted HR of 0.36 (95% CI 0.32–0.40). Further stratified analyses indicated that patients who received 1, 2, 3, 4, or ≥5 RYR prescriptions had adjusted HRs of 0.40 (95% CI 0.34–0.47), 0.39 (95% CI 0.30–0.49), 0.31 (95% CI 0.23–0.43), 0.43 (95% CI 0.31–0.60), and 0.32 (95% CI 0.27–0.38), respectively.

**Table 2:** The adjusted risk of ESRD between people with and without use of red yeast rice prescription during the follow-up period.

			Risk of ESRD			
	n	PYs	Events	Incidence†	HR	(95% CI)*
No RYR‡	90836	913286	3344	3.7	1.00	(reference)
RYR cohort‡	67876	562606	508	0.9	0.36	(0.32-0.40)
Patients used frequency of RYR						
1	20186	163096	170	1.0	0.40	(0.34-0.47)
2	9673	78403	77	1.0	0.39	(0.30-0.49)
3	6511	52980	42	0.8	0.31	(0.23-0.43)
4	4776	39181	41	1.0	0.43	(0.31-0.60)
≥5	26730	228946	178	0.8	0.32	(0.27−0.38)
Time-weighted average dose, mg/day						
<100	18894	129187	134	1.0	0.40	(0.33-0.48)
100-199	2570	23814	16	0.7	0.29	(0.18-0.48)
200-299	1933	16609	9	0.5	0.22	(0.11-0.43)
300-399	1823	14868	12	0.8	0.35	(0.20-0.63)
400-499	1801	14566	8	0.5	0.23	(0.11-0.46)
≥500	40855	297483	277	0.9	0.36	(0.32-0.41)

CI, confidence interval; CCI, Charlson comorbidity index; HR, hazard ratio; PYs, person-years; RYR, red yeast rice.

*Adjusted for all covariates listed in Table 1.

†Per 1000 person-years.

‡The mean follow-up time for RYR and non-RYR cohort were 8.3 and 10.1 years, respectively.

Stratified analyses (Fig 2) revealed that both female (HR 0.30, 95% CI 0.26–0.35) and male (HR 0.42, 95% CI 0.36–0.49) RYR users exhibited a significantly lower incidence of ESRD than non-users. Age-specific analyses showed that RYR use was associated with a reduced ESRD risk across all age groups, including individuals aged 20−29 years (HR 0.25, 95% CI 0.17–0.38) and those aged ≥70 years (HR 0.73, 95% CI 0.58–0.90).

**Fig 2:**
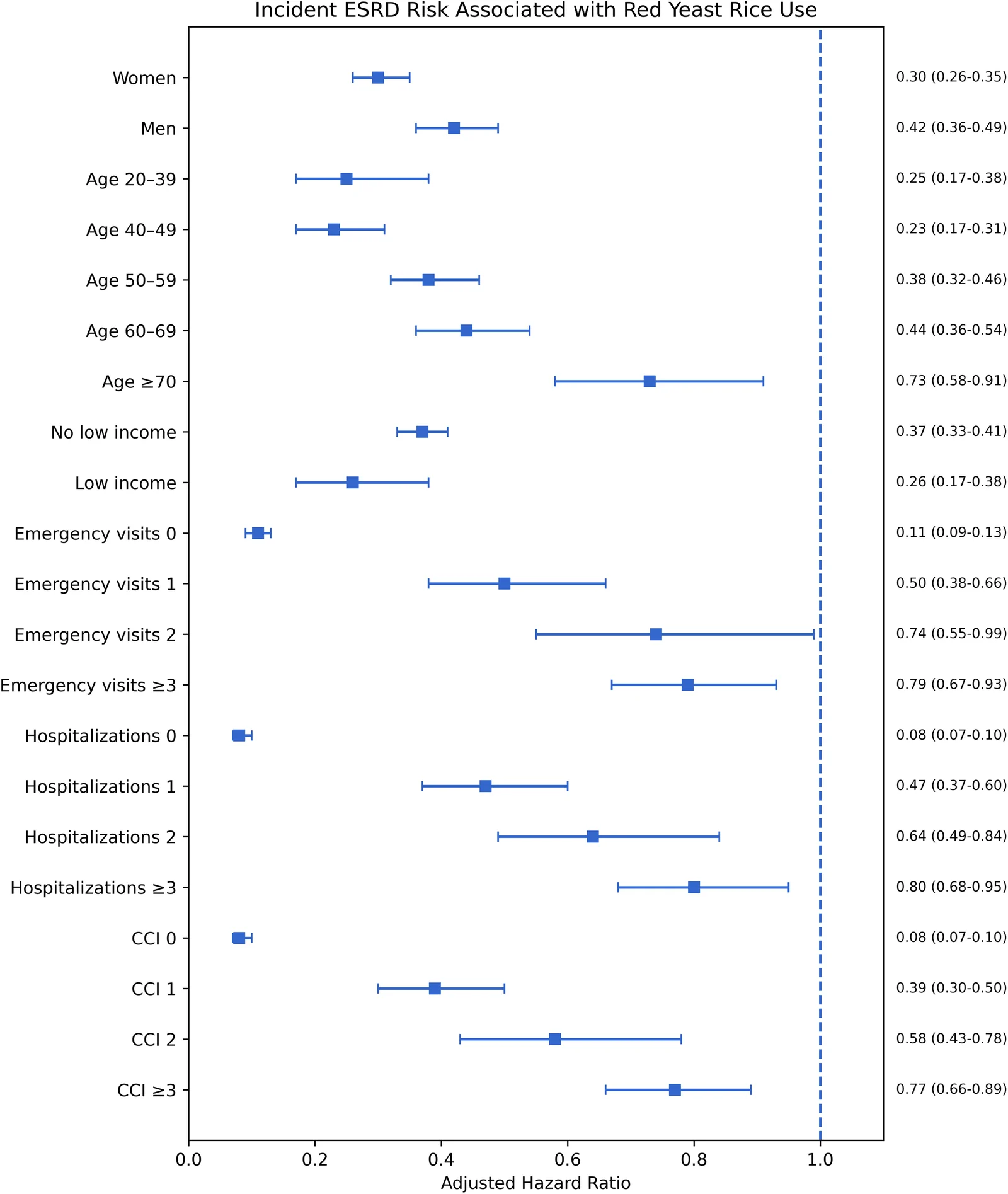
The stratified analysis for the risk of end-stage renal disease between people with and without use of red yeast rice during the follow-up period.

Similarly, the reduction in ESRD risk was observed in both low-income individuals (HR 0.26, 95% CI 0.17–0.38) and those without low-income status (HR 0.37, 95% CI 0.33–0.41). Regarding healthcare utilization, the protective effect of RYR remained consistent across emergency visit and hospitalization frequency subgroups. The greatest reduction in risk was noted among those with no emergency visits (HR 0.11, 95% CI 0.09–0.13) and no prior hospitalizations (HR 0.08, 95% CI 0.07–0.10), suggesting a particularly strong association in healthier individuals. Among participants with a Charlson Comorbidity Index score of 0, RYR users had an HR of 0.08 (95% CI 0.07–0.10) for ESRD, while those with higher comorbidity scores (≥3) still exhibited a significant but more moderate risk reduction (HR 0.77, 95% CI 0.66–0.89).

The robustness of these findings was confirmed through sensitivity analyses (Table 3). After excluding ESRD cases occurring within the first 1, 2, 3, 4, 5, and 6 months of follow-up, the adjusted HRs remained statistically significant at 0.56 (95% CI 0.50–0.63), 0.72 (95% CI 0.64–0.81), 0.72 (95% CI 0.64–0.81), 0.72 (95% CI 0.64–0.81), 0.72 (95% CI 0.64–0.81), and 0.72 (95% CI 0.64–0.81), respectively. The association persisted even after adjusting for competing risks by excluding deaths during follow-up (HR 0.44, 95% CI 0.39–0.50).

**Table 3:** Sensitivity analysis for the risk of ESRD associated with red yeast rice prescription after excluding the initial incident events and deaths (cohort study).

				Risk of ESRD			
Excluding ESRD cases during initial follow-up period		N	PYs	Events	Incidence*	HR	(95% CI)†
One month	No RYR	90309	913258	2817	3.1	1.00	(reference)
	RYR	67789	562602	421	0.7	0.56	(0.50-0.63)
Two months	No RYR	90129	913242	2637	2.9	1.00	(reference)
	RYR	67785	562602	417	0.7	0.72	(0.64-0.81)
Three months	No RYR	90113	913239	2621	2.9	1.00	(reference)
	RYR	67782	562601	414	0.7	0.72	(0.64-0.81)
Four month	No RYR	90103	913236	2611	2.9	1.00	(reference)
	RYR	67779	562600	411	0.7	0.72	(0.64-0.81)
Five months	No RYR	90094	913233	2602	2.8	1.00	(reference)
	RYR	67773	562598	405	0.7	0.72	(0.64-0.81)
Six months	No RYR	90082	913227	2590	2.8	1.00	(reference)
	RYR	67771	562597	403	0.7	0.72	(0.64-0.81)
Excluded deaths	No RYR	80144	850001	2286	2.7	1.00	(reference)
	RYR	66291	554302	411	0.7	0.44	(0.39-0.50)

CI, confidence interval; HR, hazard ratio; PYs, person-years; RYR, red yeast rice.

*Per 1000 person-years.

†Adjusted for all covariates listed in Table 1.

Further comparisons (Table 4) indicated that patients in the RYR cohort consistently exhibited a lower ESRD risk compared to those prescribed other statins, including atorvastatin (HR 0.16, 95% CI 0.15–0.17), fluvastatin (HR 0.17, 95% CI 0.16–0.19), rosuvastatin (HR 0.19, 95% CI 0.17–0.21), simvastatin (HR 0.24, 95% CI 0.21–0.26), and pravastatin (HR 0.35, 95% CI 0.32–0.39). Risk of ESRD associated with RYR use during the various follow-up period (Table 5). Table 6 presents the interaction between RYR and cardiovascular medications on the risk of ESRD during the follow-up period.

**Table 4:** The adjusted risk of incident ESRD between people with and without use of red yeast rice prescription during the follow-up period (cohort study).

			Incident ESRD			
	n	PYs	Events	Incidence†	HR	(95% CI)‡
No-RYR cohort (used Atorvastatin)	1036114	9680673	55825	5.8	1.00	(reference)
RYR cohort	67876	562606	508	0.9	0.16	(0.15-0.17)
No-RYR cohort (used Fluvastatin)	252711	2347674	12319	5.2	1.00	(reference)
RYR cohort	67876	562606	508	0.9	0.17	(0.16-0.19)
No-RYR cohort (used Rosuvastatin)	827472	7566745	41559	5.5	1.00	(reference)
RYR cohort	67876	562606	508	0.9	0.19	(0.17-0.21)
No-RYR cohort (used Simvastatin)	569819	5618855	25475	4.5	1.00	(reference)
RYR cohort	67876	562606	508	0.9	0.24	(0.21-0.26)
No-RYR cohort (used Pravastatin)	236012	2268443	8107	3.6	1.00	(reference)
RYR cohort	67876	562606	508	0.9	0.35	(0.32-0.39)

CI, confidence interval; ESRD, end-stage renal disease; HR, hazard ratio; PYs, person-years; RYR, red yeast rice.

†Per 1000 person-years.

‡Adjusted for all covariates listed in Table 1. Bonferroni correction was applied to adjust for multiple testing.

**Table 5:** Risk of ESRD (adjusted competing risk) and major renal adverse events associated with during the follow-up period (cohort study).

			Risk of ESRD			
	n	PYs	Events	Incidence	HR	(95% CI)
ESRD (adjusted competing risk)*						
No RYR	90836	913286	2187	2.4	1.00	(reference)
RYR cohort	67876	562606	378	0.7	0.44	(0.39-0.50)
Major renal adverse events†						
No RYR	90836	834677	11597	13.9	1.00	(reference)
RYR cohort	67876	496528	1676	3.4	0.67	(0.63-0.71)
During the 0–2 years of follow-up						
No RYR	90836	178883	1055	5.9	1.00	(reference)
RYR cohort	67876	135369	152	1.1	0.09	(0.08-0.11)
During the 0–4 years of follow-up						
No RYR	90836	354121	1518	4.3	1.00	(reference)
RYR cohort	67876	270041	256	0.9	0.17	(0.15-0.20)
During the 0–6 years of follow-up						
No RYR	90836	525157	2026	3.9	1.00	(reference)
RYR cohort	67876	403858	350	0.9	0.24	(0.21-0.27)
During the 0–8 years of follow-up						
No RYR	90836	691380	2566	3.7	1.00	(reference)
RYR cohort	67876	536915	422	0.8	0.28	(0.25−0.32)
During the 0–10 years of follow-up						
No RYR	90836	852883	3008	3.5	1.00	(reference)
RYR cohort	67876	669468	452	0.7	0.29	(0.26-0.32)

*Using the Fine-Gray subdistribution hazard model, which excludes all-cause death cases from the outcome events but retains them in the risk set during the follow-up period.

†Included ESRD and all-cause death.

**Table 6:** The adjusted risk of End-stage renal disease in patients received RYR by medications during the follow-up period.

				Risk of ESRD			
Use of ESRD-related medications		n	PYs	Events	Incidence†	HR	(95% CI)‡
No ACE inhibitors drug use	No RYR	59997	548063	1697	3.1	1.00	(reference)
	RYR	60351	437635	345	0.8	0.28	(0.24-0.31)
ACE inhibitors drug use	No RYR	30839	286614	1411	4.9	1.00	(reference)
	RYR	7525	58893	111	1.9	0.56	(0.46-0.69)
No ARBs drug use	No RYR	31414	281806	849	3.0	1.00	(reference)
	RYR	42213	304985	142	0.5	0.10	(0.09-0.12)
ARBs drug use	No RYR	59422	552871	2259	4.1	1.00	(reference)
	RYR	25663	191543	314	1.6	0.66	(0.58-0.74)
No Nephrotoxic drugs use	No RYR	67042	616005	2317	3.8	1.00	(reference)
	RYR	58926	428134	372	0.9	0.30	(0.26-0.33)
Nephrotoxic drugs use	No RYR	23794	218672	791	3.6	1.00	(reference)
	RYR	8950	68394	84	1.2	0.71	(0.56-0.90)
No diuretics drugs use	No RYR	45846	429479	786	1.8	1.00	(reference)
	RYR	53953	391466	127	0.3	0.08	(0.07-0.10)
Diuretics drugs use	No RYR	44990	405198	2322	5.7	1.00	(reference)
	RYR	13923	105062	329	3.1	0.71	(0.63-0.80)

CI, confidence interval; HR, hazard ratio; PYs, person-years; RYR, red yeast rice.

†Per 1000 person-years.

‡Adjusted for all covariates listed in Table 1.

S1 Fig. presents the Kaplan-Meier curves for ESRD-free survival according to RYR use (log-rank test, p < 0.0001). S1 Table showed the ICD-9-CM codes, ICD-10-CM codes, procedure codes, and physician’s order codes for medical diseases and treatment procedures. Baseline characteristics of the propensity score-matched cohorts with and without RYR use are presented in S2 Table. During the follow-up period, the adjusted risk of ESRD between the two cohorts was evaluated after propensity-score matching (S3 Table). Furthermore, the adjusted risk of ESRD associated with RYR use was analyzed according to different follow-up time periods (S4 Table).

## Discussion

The present investigation represents the primary research to explore the link between RYR prescriptions and a decreased probability of ESRD among hyperlipidemia patients over an extended follow-up timeframe. By analyzing real-world evidence from a dependable insurance claims database, we observed that the consumption of RYR consistently correlated with a lower risk of ESRD across all analyzed subgroups. This finding was further supported by sensitivity analyses and the identification of a dose-response effect. Our observations also indicated that patients with ESRD who utilized RYR did not exhibit a higher frequency of adverse complications compared to the non RYR group during the initial follow-up period.

Earlier publications have suggested that the administration of statins might elevate the probability of renal disease or functional impairment in the kidneys [24]. In contrast, a research project conducted in the Netherlands showed that prolonged statin therapy among individuals with peripheral arterial disease was linked to better cardiovascular health and a delayed progression toward ESRD [25]. Supporting this view, a meta analysis revealed that statins may be effective in lowering levels of albuminuria or proteinuria, especially for those suffering from cardiovascular conditions [26]. Furthermore, an investigation involving patients with peripheral arterial disease and hyperlipidemia noted a reduction in creatinine concentrations following 3–4 months of simvastatin use, a benefit that appeared regardless of changes in LDL cholesterol [27]. Consequently, our findings regarding the association between RYR consumption and a decreased risk of ESRD compared to lovastatin are consistent with these previous observations.

We suggest three mechanisms to explain why RYR use correlates with a decreased ESRD risk. First, it is well-established that inflammation acts as a major catalyst for kidney dysfunction, facilitating the progression toward ESRD and increasing death rates [28–30]. Because RYR has demonstrated the capacity to modulate lipid profiles and reduce hs-CRP concentrations [31], it may offer clinical benefits by protecting the kidneys from inflammation-mediated deterioration in patients with dyslipidemia.

Second, renal impairment is profoundly influenced by atherosclerosis, particularly regarding proteinuria onset in diabetic cohorts [32]. Evidence suggests that systemic atherosclerotic burden is a critical determinant of ESRD risk across diverse populations [33]. This vascular deterioration is tightly linked to CKD progression and correlates with elevated baseline levels of uric acid, phosphate, and atheroma plaques [34]. While dyslipidemia is a recognized driver of CKD, statin use has not consistently shown a preventative effect in hypercholesterolemic individuals with preserved kidney function [35]. Therefore, lipid-lowering strategies should be personalized based on a patient’s cardiovascular risk profile [36]. By effectively managing dyslipidemia, it may be possible to decelerate the transition from nephrotic syndrome to ESRD [37]. Similarly, RYR might offer renoprotection by lowering total cholesterol, LDL-C, and triglycerides, thereby reducing the atherosclerotic burden and delaying the functional decline of the kidneys.

Third, the socio-behavioral characteristics of TCM users in Taiwan may influence our findings. TCM is frequently integrated as a complementary therapeutic modality, and patients who seek such integrated care often demonstrate higher health literacy and a more proactive stance toward preventive health. Established research indicates that TCM utilization is linked to healthier lifestyle choices and a more positive health attitude [38,39]. Furthermore, demographic trends suggest that individuals with higher educational attainment or professional status (white-collar) are more predisposed to use TCM [38,39]. Consequently, it is possible that the lower ESRD risk observed among RYR users is partially attributable to these health-seeking behaviors and overall lifestyle management, rather than solely the pharmacological effects of the supplement.

RYR use was consistently associated with a significantly lower risk of ESRD across all examined subgroups, including sex, seven age groups, income, hospitalisation frequency, emergency visit frequency, Charlson Comorbidity Index scores, and five statin comparators. The association between RYR use and a reduced risk of ESRD was more pronounced in individuals who were younger, had no comorbidities, and had fewer healthcare visits than in those with multiple comorbidities or frequent healthcare utilization (more than three emergency visits or hospitalizations). A plausible explanation is that the beneficial effects of RYR may be masked or attenuated by more severe underlying medical conditions. Similar findings have been reported in previous studies [10–12,15].

Continuous surveillance of the safety profile of RYR remains imperative for clinical practice. Given the extensive adoption of RYR for dyslipidemia management, a rigorous appraisal of its safety is is of paramount importance. While some clinical reports have linked RYR to adverse events such as myopathy, hepatic impairment, and erectile dysfunction [40–42], these findings contrast with certain statin reviews suggesting potential benefits for erectile health [43]. Interestingly, our earlier findings indicated a protective association between RYR and liver cirrhosis risk [15]. Current evidence generally supports the tolerability of RYR, positioning it as a viable option for statin-intolerant populations [19]. Nevertheless, we advocate for systematic monitoring of renal (e.g., eGFR, creatinine) and hepatic (e.g., AST, ALT) biomarkers, alongside clinical screening for muscular or sexual dysfunction. Distinct from over-the-counter supplements, our analysis focuses on physician-prescribed RYR (LipoCol Forte^®^). This formulation is manufactured under stringent Good Manufacturing Practices, ensuring superior stability and pharmacological consistency.

Puberulic acid is the key contaminant in the RYR food safety incident involving Japan’s Kobayashi Pharmaceutical. It is not a metabolite of the RYR mold itself but rather a byproduct produced by Penicillium that was accidentally introduced during the fermentation process. Several case reports have documented Fanconi syndrome in affected individuals, suggesting that functional foods contaminated with nephrotoxins may induce the syndrome via acute tubular necrosis [1–5,44]. RYR lowers lipids primarily by inhibiting HMG-CoA reductase via Monacolin K, while simultaneously reducing pro inflammatory cytokines such as IL-6 and TNF-alpha to mitigate metabolic risk. When combined with Morus alba, these compounds improve endothelial function and insulin signaling, offering a synergistic and multifaceted approach to cardiometabolic health [45]. Previous research suggests that the superior lipid-lowering effect of LopiGLIK^®^ compared to Armolipid Plus® cannot be explained by the marginal difference in Monacolin K dosage (3.3 mg vs. 2.99 mg). Instead, other ingredients such as Morus alba likely play a key role in enhancing total cholesterol reduction [46]. While both lovastatin and RYR effectively improve the gut microbiota, RYR offers a safer alternative to pure lovastatin. Although they share core lipid metabolic pathways, the presence of polysaccharides and other natural compounds in RYR uniquely influences circadian and estrogen signaling pathways. This multifaceted composition contributes to the enhanced safety profile of RYR over conventional lovastatin treatments [47]

Lovastatin has been integrated into the National Health Insurance system in Taiwan since its inception and has long been established as the first-line standard of care for hyperlipidemia. RYR (LipoCol Forte^®^) was introduced into the National Health Insurance reimbursement scheme in 2010. Although TCM enjoys a high annual utilization rate in Taiwan (approximately 30%) [38,39], it is often utilized as a complementary rather than a primary treatment for chronic metabolic diseases. Lovastatin is a regulated pharmaceutical often prescribed to patients with significantly high total cholesterol or established cardiovascular risk. A significant portion of RYR users choose the supplement because they experienced side effects (such as myalgia) from previous statin use. These patients may have different underlying muscle or metabolic sensitivities compared to those who tolerate lovastatin well.

Historically, statin users tend to be older and have a higher prevalence of comorbidities (such as hypertension and diabetes). RYR users are often younger with fewer systemic diseases, making them inherently lower risk for ESRD regardless of the treatment. RYR users often exhibit a “healthy user bias”. These individuals are typically more health-conscious, more likely to exercise, and more adherent to dietary restrictions than the general patient population. In the statistical analysis process, we used multiple Cox proportional models to adjust for all covariates listed in Table 1 (baseline characteristics). However, we could not control or adjust for all potential confounding factors because certain information was unavailable in the National Health Insurance database. Residual confounding is always possible in this study.

Our research is subject to several constraints. First, utilizing insurance claims to identify RYR prescriptions does not ensure that patients followed their prescribed regimens. Due to the observational design, we were unable to quantify the actual dose of RYR consumed or provide a precise assessment of medication adherence for both RYR and statins. We acknowledge that potential exposure misclassification is a limitation of this study. However, such misclassification is likely non-differential, which would typically bias the results toward the null, suggesting our findings may be a conservative estimate. Second, the absence of detailed sociodemographic (including education level and occupation) and lifestyle data (including smoking status, alcohol consumption, and physical activity) limited our capacity for a more comprehensive analysis. The information regarding physical test (such as body mass index), biochemical data (such as lipid profiles, serum creatinine or eGFR), and clinical examinations (such as X-ray, carotid sonography, and CT scan) was unavailable in this health insurance claims database. Thus, we could not determine the precise CKD stage. The influence of unmeasured confounding factors such as those listed above cannot be entirely excluded, potentially leading to residual confounding. Finally, the retrospective nature of this cohort study prevents the establishment of a definitive causal relationship between RYR use and a lower risk of ESRD. Unlike the evidence derived from randomized controlled trials, retrospective findings are inherently restricted in their ability to demonstrate direct cause and effect.

In conclusion, our findings suggest that people who used RYR had a lower risk of ESRD compared with those who used lovastatin, and this association was consistently observed across all subgroups. Nevertheless, these results must be interpreted with caution due to inherent constraints such as uncertain medication adherence and the potential for unmeasured confounding variables. The generalisability of these findings may be limited, as this study only included individuals prescribed LipoCol Forte^®^. Therefore, the results may not be applicable to other RYR-containing nutraceuticals or health supplements. To confirm these observations and delineate a clear causal link, further investigation through randomized controlled trials is essential to evaluate the therapeutic role of RYR in preventing the progression of renal disease.

## Supporting information

S1 Fig Kaplan-Meier Curves for ESRD-Free Survival According to Red Yeast Rice Use (log-rank test, p < 0.0001). (TIF)

S1 Table ICD-9-CM codes, ICD-10-CM codes, procedure codes, and physician’s order codes for medical diseases and treatment procedures. (DOC)

S2 Table Baseline characteristics between cohorts with and without use of red yeast rice prescription (cohort study with propensity-score matching). (DOC)

S3 Table The adjusted risk of ESRD between people with and without use of red yeast rice prescription during the follow-up period (cohort study with propensity-score matching). (DOC)

S4 Table The adjusted risk of ESRD between people with and without use of red yeast rice prescription during the follow-up period (cohort study with propensity-score matching). (DOC)

## Abbreviations

CI: confidence interval
ESRD: end-stage renal disease
HR: hazard ratio
ICD-9-CM: International Classification of Diseases, 9th Revision, Clinical Modification
RYR: red yeast rice
