# Supplementary material for: Does receiving red yeast rice prescriptions (LipoCol Forte®) increase the risk of end-stage renal disease or not? A retrospective cohort study
Source: PLoS One. 2026 Oct 27;21(10):e0345243. doi: 10.1371/journal.pone.0345243 (PMC13712715; doi:10.1371/journal.pone.0345243)
Supplement: Supplementary file 1 [file pone.0345243.s002.doc]

| **S1 Table.** ICD-9-CM codes, ICD-10-CM codes, procedure codes, and physician’s order codes for medical diseases and treatment procedures | |
| --- | --- |
|  | ICD-9-CM, ICD-10-CM, procedure codes, and physician’s order code |
| ESRD | ICD-9-CM 585.6 and ICD-10-CM N18.6 |
| Renal dialysis | procedure codes (included cure-item: D8 and D9) |
| Renal dialysis | physician’s order code (58001C, 58002C, 58009B, 58010A, 58010B, 58011A, 58011AB, 58011B, 58011C, 58012A, 58012B, 58013C, 58014C, 58017B, 58017C, 58018C, 58019C, 58020C, 58021C, 58022C, 58023C, 58024C, 58025C, 58026C, 58027C, 58028C, 58029C, 58030B, 69006C, P8701C, P8701C |
| AMI | ICD-9-CM: 410; ICD-10-CM: I21, I22 |
| Hypertension | ICD-9-CM: 401-405; ICD-10-CM: I10, I11, I12, I13, I15, N26.2 |
| Diabetes | ICD-9-CM: 250; ICD-10-CM: E08-E11, E13 |
| Hyperlipidemia | ICD-9-CM: 272.0-272.4; ICD-10-CM: E78.0-E78.2, E78.4, E78.5 |
| Mental disorders | ICD-9-CM: 290-319  ICD-10-CM: F01-F07, F09-F25, F28-F34, F39-F45, F48, F50-F55, F59, F60, F63-F66, F68-F73, F78-F82, F84, F88-F91, F93-F95, F98, F99, R37, G44, G47, R45.1, R45.7, R48.0, R45.86, F43.21, R41.82, R45.81, R45.82, H93.25, Z87.890, R41.840, R41.841, R41.843, R41.844 |
| Heart failure | ICD-9-CM: 428; ICD-10-CM: I50 |
| Ischemic heart disease | ICD-9-CM: 410-414  ICD-10-CM: I20.0, I20.1, I20.8, I20.9, I21.3, I21.4, I22.0, I22.1, I22.2, I22.8, I22.9, I24.0, I24.1, I24.8, I24.9, I25.2, I25.3, I25.5, I25.6, I25.9, I21.01, I21.02, I21.09, I21.11, I21.19, I21.21, I21.29, I25.10, I25.41, I25.42, I25.89, I25.110, I25.111, I25.118, I25.119, I25.700, I25.701, I25.708, I25.709, I25.710, I25.711, I25.718, I25.719, I25.720, I25.721, I25.728, I25.729, I25.730, I25.731, I25.738, I25.739, I25.750, I25.751, I25.758, I25.759, I25.760, I25.761, I25.768, I25.769, I25.790, I25.791, I25.798, I25.799, I25.810, I25.811, I25.812 |
| COPD | ICD-9-CM: 491, 492, 496  ICD-10-CM: J42, J41.0, J41.1, J41.8, J43.0, J43.1, J43.2, J43.8, J43.9, J44.0, J44.1, J44.9 |
| Liver cirrhosis | ICD-9-CM: 571.2, 571.5, 571.6  ICD-10-CM: K74 |
| Stroke | ICD-9-CM: 430, 431, 432, 433, 434, 435, 436, 437, 438  ICD-10-CM: I60, I61, I62, I63, I65, I66, I67, I68, I69, G45, G46 |
| Pneumonia | ICD-9-CM: 480, 481, 482, 483, 484, 485, 486  ICD-10-CM: J13, J14, J17, J12.0, J12.1, J12.2, J12.3, J12.9, J15.0, J15.1, J15.3, J15.4, J15.5, J15.6, J15.7, J15.8, J15.9, J16.0, J16.8, J18.0, J18.1, J18.8, J18.9, A22.1, A48.1, B25.0, B44.0, J12.81, J12.89, J15.20, J15.29, A37.91, J15.211 |
| Acute renal failure | ICD-9-CM: 584; ICD-10-CM: N17.0, N17.1, N17.2, N17.8, N17.9 |
| Pulmonary embolism | ICD-9-CM: 415  ICD-10-CM: I26.09, I26.90, I26.92, I26.99, I27.82, T80.0XXA, T81.718A, T81.72XA, T82.817A, T82.818A |
| Deep wound infection | ICD-9-CM: 958  ICD-10-CM: T79.0XXA, T79.1XXA, T79.2XXA, T79.8XXA, T79.4XXA, T79.5XXA, T79.6XXA, T79.7XXA, T79.9XXA, T79.A0XA, T79.A11A, T79.A12A, T79.A19A, T79.A21A, T79.A22A, T79.A29A, T79.A3XA, T79.A9XA |
| Septicemia | ICD-9-CM: 038, 998.5  ICD-10-CM: A40.0, A40.1, A40.3, A40.8, A40.9, A41.1, A41.2, A41.3, A41.4, A41.9, R65.10, R65.11, R65.20, A41.01, A41.02, A41.50, A41.51, A41.52, A41.53, A41.59, A41.81, A41.89, K68.11, R50.84, T81.4XXA |
| Postoperative bleeding | ICD-9-CM: 998.0, 998.1, 998.2  ICD-10-CM: D78.01, D78.02, D78.21, D78.22, E36.01, E36.02, G97.31, G97.32, G97.51, G97.52, H95.21, H95.22, H95.41, H95.42, I97.42, I97.62, J95.61, J95.62, K91.61, K91.62, L76.01, L76.02, L76.21, L76.22, N99.61, N99.62, D78.01, D78.02, D78.21, D78.22, E36.01, E36.02, G97.31, G97.32, G97.51, G97.52, H95.21, H95.22, H95.41, H95.42, I97.42, I97.62, J95.61, J95.62, K91.61, K91.62, L76.01, L76.02, L76.21, L76.22, N99.61, N99.62, D78.11, D78.12, E36.11, E36.12, G96.11, G97.41, G97.48, G97.49, H95.31, H95.32, I97.51, I97.52, J95.71, J95.72, K91.71, K91.72, L76.11, L76.12, N99.71, N99.72, H59.111, H59.112, H59.113, H59.119, H59.121, H59.122, H59.123, H59.129, H59.311, H59.312, H59.313, H59.319, H59.321, H59.322, H59.323, H59.329, I97.410, I97.411, I97.418, I97.610, I97.611, I97.618, J95.830, J95.831, K91.840, K91.841, M96.810, M96.811, M96.830, M96.831, N99.820, N99.821, H59.111, H59.112, H59.113, H59.119, H59.121, H59.122, H59.123, H59.129, H59.311, H59.312, H59.313, H59.319, H59.321, H59.322, H59.323, H59.329, I97.410, I97.411, I97.418, I97.610, I97.611, I97.618, J95.830, J95.831, K91.840, K91.841, M96.810, M96.811, M96.830, M96.831, N99.820, N99.821, H59.211, H59.212, H59.213, H59.219, H59.221, H59.222, H59.223, H59.229, M96.820, M96.821, T81.10XA, T81.11XA, T81.12XA, T81.19XA, T88.8XXA |
| Urinary tract infection | ICD-9-CM: 599.0; ICD-10-CM: N39.0 |
|  | |
