# Supplementary material for: Does receiving red yeast rice prescriptions (LipoCol Forte®) increase the risk of end-stage renal disease or not? A retrospective cohort study
Source: PLoS One. 2026 Oct 27;21(10):e0345243. doi: 10.1371/journal.pone.0345243 (PMC13712715; doi:10.1371/journal.pone.0345243)
Supplement: Supplementary file 2 [file pone.0345243.s003.doc]

| **S2 Table. Baseline characteristics between cohorts with and without use of red yeast rice prescription (cohort study with propensity-score matching)** | | | | | |
| --- | --- | --- | --- | --- | --- |
|  | No RYR  N=21881 | | RYR prescription  N=21881 | | p-value |
| Sex | n | (%) | n | (%) | 1.0000 |
| Female | 12982 | (59.3) | 12982 | (59.3) |  |
| Male | 8899 | (40.7) | 8899 | (40.7) |  |
| Age, years |  |  |  |  | 1.0000 |
| 20-29 | 302 | (1.4) | 302 | (1.4) |  |
| 30-39 | 1303 | (6.0) | 1303 | (6.0) |  |
| 40-49 | 4304 | (19.7) | 4304 | (19.7) |  |
| 50-59 | 8870 | (40.5) | 8870 | (40.5) |  |
| 60-69 | 5244 | (24.0) | 5244 | (24.0) |  |
| 70-79 | 1651 | (7.6) | 1651 | (7.6) |  |
| ≥80 | 207 | (1.0) | 207 | (1.0) |  |
| Low income |  |  |  |  | 1.0000 |
| No | 21699 | (99.2) | 21699 | (99.2) |  |
| Yes | 182 | (0.8) | 182 | (0.8) |  |
| Number of hospitalizations |  |  |  |  | 1.0000 |
| 0 | 13387 | (61.2) | 13387 | (61.2) |  |
| 1 | 4116 | (18.8) | 4116 | (18.8) |  |
| 2 | 1371 | (6.3) | 1371 | (6.3) |  |
| ≥3 | 3007 | (13.7) | 3007 | (13.7) |  |
| Number of emergency visits |  |  |  |  | 1.0000 |
| 0 | 8687 | (39.7) | 8687 | (39.7) |  |
| 1 | 4743 | (21.7) | 4743 | (21.7) |  |
| 2 | 2355 | (10.8) | 2355 | (10.8) |  |
| ≥3 | 6096 | (27.9) | 6096 | (27.9) |  |
| Medical conditions |  |  |  |  |  |
| Hypertension | 10249 | (46.8) | 10249 | (46.8) | 1.0000 |
| Diabetes | 7334 | (33.5) | 7334 | (33.5) | 1.0000 |
| Mental disorders | 7957 | (36.4) | 7957 | (36.4) | 1.0000 |
| COPD | 934 | (4.3) | 934 | (4.3) | 1.0000 |
| Ischemic heart disease | 2607 | (11.9) | 2607 | (11.9) | 1.0000 |
| Heart failure | 279 | (1.3) | 279 | (1.3) | 1.0000 |
| Liver cirrhosis | 713 | (3.3) | 713 | (3.3) | 1.0000 |
| CCI, score |  |  |  |  | 1.0000 |
| 0 | 5015 | (22.9) | 5015 | (22.9) |  |
| 1 | 4639 | (21.2) | 4639 | (21.2) |  |
| 2 | 3067 | (14.0) | 3067 | (14.0) |  |
| ≥3 | 9160 | (41.9) | 9160 | (41.9) |  |
| Anticoagulant drug use |  |  |  |  | 1.0000 |
| No | 21726 | (99.3) | 21726 | (99.3) |  |
| Yes | 155 | (0.7) | 155 | (0.7) |  |
| Anti-hypertension drug use |  |  |  |  | 1.0000 |
| No | 15340 | (70.1) | 15340 | (70.1) |  |
| Yes | 6541 | (29.9) | 6541 | (29.9) |  |
| ACE inhibitors drug use |  |  |  |  | 1.0000 |
| No | 19311 | (88.3) | 19311 | (88.3) |  |
| Yes | 2570 | (11.8) | 2570 | (11.8) |  |
| ARBs drug use |  |  |  |  | 1.0000 |
| No | 11596 | (53.0) | 11596 | (53.0) |  |
| Yes | 10285 | (47.0) | 10285 | (47.0) |  |
| Nephrotoxic drugs use |  |  |  |  | 1.0000 |
| No | 19413 | (88.7) | 19413 | (88.7) |  |
| Yes | 2468 | (11.3) | 2468 | (11.3) |  |
| Diuretics drugs use |  |  |  |  | 1.0000 |
| No | 17294 | (79.0) | 17294 | (79.0) |  |
| Yes | 4587 | (21.0) | 4587 | (21.0) |  |
| CCI, Charlson comorbidity index; RYR, red yeast rice | | | | | |
