# Supplementary material for: Does receiving red yeast rice prescriptions (LipoCol Forte®) increase the risk of end-stage renal disease or not? A retrospective cohort study
Source: PLoS One. 2026 Oct 27;21(10):e0345243. doi: 10.1371/journal.pone.0345243 (PMC13712715; doi:10.1371/journal.pone.0345243)
Supplement: Supplementary file 3 [file pone.0345243.s004.doc]

| **S3 Table. The adjusted risk of ESRD between people with and without use of red yeast rice prescription during the follow-up period (cohort study with propensity-score matching)** | | | | | | |
| --- | --- | --- | --- | --- | --- | --- |
|  |  |  | Risk of ESRD | | | |
|  | n | PYs | Events | Incidence† | HR | (95% CI)* |
| No RYR‡ | 21881 | 200923 | 705 | 3.5 | 1.00 | (reference) |
| RYR cohort‡ | 21881 | 158193 | 178 | 1.1 | 0.27 | (0.23-0.32) |
| Patients used frequency of RYR |  |  |  |  |  |  |
| 1 | 6363 | 44680 | 57 | 1.3 | 0.29 | (0.22-0.37) |
| 2 | 3087 | 21664 | 28 | 1.3 | 0.31 | (0.22-0.46) |
| 3 | 2049 | 14461 | 15 | 1.0 | 0.25 | (0.15-0.42) |
| 4 | 1539 | 10874 | 17 | 1.6 | 0.39 | (0.24-0.63) |
| ≥5 | 8843 | 66515 | 61 | 0.9 | 0.23 | (0.18-0.30) |
| CI, confidence interval; CCI, Charlson comorbidity index; HR, hazard ratio; PYs, person-years; RYR, red yeast rice  *Adjusted for all covariates listed in Table S5.  †Per 1000 person-years  ‡The mean follow-up time for RYR and non-RYR cohort were 7.2 and 9.2 years, respectively. | | | | | | |
