# Supplementary material for: Does receiving red yeast rice prescriptions (LipoCol Forte®) increase the risk of end-stage renal disease or not? A retrospective cohort study
Source: PLoS One. 2026 Oct 27;21(10):e0345243. doi: 10.1371/journal.pone.0345243 (PMC13712715; doi:10.1371/journal.pone.0345243)
Supplement: Supplementary file 4 [file pone.0345243.s005.doc]

| **S4 Table. The adjusted risk of ESRD between people with and without use of red yeast rice prescription during the follow-up period (cohort study with propensity-score matching)** | | | | | | | |
| --- | --- | --- | --- | --- | --- | --- | --- |
|  | |  |  | Risk of ESRD | | | |
| Time period of follow-up | | n | PYs | Events | Incidence† | HR | (95% CI)‡ |
| 0~2 years | No RYR | 21881 | 42731 | 496 | 11. | 1.00 | (reference) |
|  | RYR | 21881 | 43627 | 59 | 1.4 | 0.10 | (0.08-0.13) |
| 0~4 years | No RYR | 21881 | 85174 | 532 | 6.2 | 1.00 | (reference) |
|  | RYR | 21881 | 87032 | 97 | 1.1 | 0.16 | (0.13-0.20) |
| 0~6 years | No RYR | 21881 | 127321 | 583 | 4.6 | 1.00 | (reference) |
|  | RYR | 21881 | 130150 | 128 | 1.0 | 0.20 | (0.16-0.24) |
| 0~8 years | No RYR | 21881 | 169086 | 631 | 3.7 | 1.00 | (reference) |
|  | RYR | 21881 | 172941 | 163 | 0.9 | 0.23 | (0.20-0.28) |
| 0~10 years | No RYR | 21881 | 210430 | 689 | 3.3 | 1.00 | (reference) |
|  | RYR | 21881 | 215484 | 176 | 0.8 | 0.23 | (0.20-0.28) |
| CI, confidence interval; CCI, Charlson comorbidity index; HR, hazard ratio; PYs, person-years; RYR, red yeast rice  *Adjusted for all covariates listed in Table S5.  †Per 1000 person-years | | | | | | | |
